# Plasma GDF-15 Levels Are Associated With HIV Reservoir Markers Independently of Inflammation in People With HIV on Antiretroviral Therapy

**DOI:** 10.1093/infdis/jiag202

**Published:** 2026-04-09

**Authors:** Stephane Isnard, Léna Royston, Carolina A Berini, Tsoarello Mabanga, Abdulrahman Al-abdulmalek, Orthy Aiyana, Liqin Sun, Mohamed El-Far, Amélie Pagliuzza, Delphine Planas, Simeng Bu, Meetinder Kaur Pardesi, Rémi Fromentin, Petronela Ancuta, Madeleine Durand, Nicolas Chomont, Jean-Pierre Routy

**Affiliations:** Research Institute, McGill University Health Centre, Montréal, Québec, Canada; Chronic Viral Illness Service, McGill University Health Centre, Montréal, Québec, Canada; Research Institute, McGill University Health Centre, Montréal, Québec, Canada; Chronic Viral Illness Service, McGill University Health Centre, Montréal, Québec, Canada; Division of Infectious Diseases, Geneva University Hospitals, Geneva, Switzerland; Research Institute, McGill University Health Centre, Montréal, Québec, Canada; Chronic Viral Illness Service, McGill University Health Centre, Montréal, Québec, Canada; Research Institute, McGill University Health Centre, Montréal, Québec, Canada; Chronic Viral Illness Service, McGill University Health Centre, Montréal, Québec, Canada; Research Institute, McGill University Health Centre, Montréal, Québec, Canada; Division of Hematology, McGill University Health Centre, Montréal, Québec, Canada; Research Institute, McGill University Health Centre, Montréal, Québec, Canada; Chronic Viral Illness Service, McGill University Health Centre, Montréal, Québec, Canada; Research Institute, McGill University Health Centre, Montréal, Québec, Canada; Chronic Viral Illness Service, McGill University Health Centre, Montréal, Québec, Canada; National Clinical Research Center for Infectious Diseases, Shenzhen Third People's Hospital, Shenzhen, China; Centre de Recherche du Centre Hospitalier de l’Université de Montréal,Montréal, Québec, Canada; Centre de Recherche du Centre Hospitalier de l’Université de Montréal,Montréal, Québec, Canada; Centre de Recherche du Centre Hospitalier de l’Université de Montréal,Montréal, Québec, Canada; Research Institute, McGill University Health Centre, Montréal, Québec, Canada; Chronic Viral Illness Service, McGill University Health Centre, Montréal, Québec, Canada; Research Institute, McGill University Health Centre, Montréal, Québec, Canada; Chronic Viral Illness Service, McGill University Health Centre, Montréal, Québec, Canada; Centre de Recherche du Centre Hospitalier de l’Université de Montréal,Montréal, Québec, Canada; Centre de Recherche du Centre Hospitalier de l’Université de Montréal,Montréal, Québec, Canada; Département de Microbiologie, Infectiologie et Immunologie, Faculté de Médecine, Université de Montréal, Montréal, Québec, Canada; Département de Microbiologie, Infectiologie et Immunologie, Faculté de Médecine, Université de Montréal, Montréal, Québec, Canada; Département de Médecine, Faculté de Médecine, Université de Montréal, Montréal, Québec, Canada; Centre de Recherche du Centre Hospitalier de l’Université de Montréal,Montréal, Québec, Canada; Research Institute, McGill University Health Centre, Montréal, Québec, Canada; Chronic Viral Illness Service, McGill University Health Centre, Montréal, Québec, Canada; Division of Hematology, McGill University Health Centre, Montréal, Québec, Canada

**Keywords:** GDF-15, HIV reservoirs, ART, inflammation, CD4 T cells

## Abstract

**Background:**

People with human immunodeficiency virus (HIV) receiving antiretroviral therapy (ART) have increased risks of non-AIDS comorbidities. Growth differentiation factor 15 (GDF-15) is a mitokine released upon mitochondrial stress and is a validated aging biomarker. Herein, we assessed associations between plasma GDF-15 levels, inflammation, and HIV reservoir markers in ART-treated people with HIV (PWH).

**Methods:**

Blood samples were collected from 78 ART-naive, 140 ART-treated PWH (median ART duration, 15.6 years) and 83 uninfected control participants. GDF-15 and markers of inflammation were quantified in plasma by enzyme-linked immunosorbent assay (ELISA) and multiplex assays. Integrated HIV DNA levels were measured in isolated CD4 T cells by ultrasensitive quantitative Alu polymerase chain reaction. Intracellular GDF-15 production was assessed ex vivo by flow cytometry, and in supernatants by ELISA after in vitro stimulations.

**Results:**

Plasma GDF-15 levels were higher in ART-treated PWH compared to ART-naive PWH or controls, independently of age. Ex vivo, GDF-15 was produced by classical, intermediate, and nonclassical monocytes, but absent in T cells, B cells, natural killer cells, and dendritic cells. Plasma and monocyte intracellular GDF-15 levels correlated strongly (*r* = 0.96, *P* = .002). Plasma GDF-15 levels did not correlate with the majority of inflammatory markers quantified in plasma. Conversely, plasma GDF-15 levels correlated with the validated non-AIDS inflammatory comorbidity marker suPAR (soluble urokinase plasminogen activator receptor; *r* = 0.59, *P* < .001), as well as with integrated HIV DNA levels in CD4^+^ T cells (*r* = 0.47, *P* < .01).

**Conclusions:**

Plasma levels of GDF-15, mainly produced by monocytes, were positively associated with HIV reservoir markers and coincided with increased level of suPAR, suggesting that HIV persistence is associated with increased mitochondrial stress and comorbidity risks in ART-treated PWH.

## Teaser

Plasma GDF-15 levels were higher in ART-treated PWH compared to ART-naive or HIV-negative controls. Monocyte/macrophage production of GDF-15 correlated with markers of HIV reservoir size and non-AIDS comorbidities in ART-treated PWH, suggesting a participation of tissue damage in HIV persistence.

Antiretroviral therapy (ART), by controlling human immunodeficiency virus-1 (HIV-1) replication, prevents AIDS and secondary transmission. However people with HIV (PWH) present higher risks for the development of non-AIDS comorbidities such as cardiovascular disease, metabolic complications, and cancer [1, 2]. Persistent inflammation is hypothesized to contribute to the increased risk of non-AIDS comorbidities in PWH on long-term ART. Inflammation and higher risk of comorbidities have been linked with accelerated and accentuated aging, a phenomenon indicating early and greater risk of age-linked comorbidities in PWH [3, 4].

ART does not lead to the eradication of HIV, since latent HIV genomes persist in long-lived cells called HIV reservoirs. The persistence of HIV genomes in CD4 T cells and tissue macrophages represents the main barrier to an HIV cure. A fraction of the HIV reservoir is transcriptionally active, and this residual and continuous production of HIV RNA and proteins contribute to T-cell activation and immune dysregulation [5, 6].

Growth differentiation factor 15 (GDF-15), also called macrophage inhibitory cytokine 1 (MIC-1), is a member of the transforming growth factor beta (TGF-β) family [7]. GDF-15 is normally expressed at low levels in most organs, whereas its production increases during tissue stress and injury and with aging [8–10]. GDF-15 is notably produced upon mitochondrial stress and is considered an integrative marker of biological stress [11]. GDF-15 has been associated with a regulatory phenotype and tolerogenic responses [12].

Circulating GDF-15 levels are elevated in people with cardiovascular disease, including in PWH on ART [13]. Similarly, high levels of this mitokine have been reported in people with COVID-19 [14, 15], several cancers [16], and other comorbidities [17–20]. GDF-15 reduces appetite by binding to its receptor GDNF family receptor alpha-like (GFRAL) in the brainstem, and metformin-associated weight loss is linked with induction of GDF-15 [17, 18, 21].

Herein, we compared the levels of GDF-15 in the plasma of PWH on ART with age-matched control participants, and evaluated factors associated with GDF-15 elevation. We then examined associations between GDF-15 levels and HIV persistence markers.

## MATERIALS AND METHODS

### Study Design and Population

This cross-sectional and longitudinal study used samples and data from adult participants from the Montreal Primary HIV infection cohort, the Canadian HIV and Aging Cohort Study (CHACS), which study design and protocol have been previously reported [22], as well as the HIV pathogenesis biobank. In the CHACS cohort, PWH over the age of 40 years or having lived with HIV for at least 15 years were recruited in HIV medical centers, with the majority being men who have sex with men. HIV-uninfected controls aged ≥40 years were recruited at the same medical centers. Through the HIV pathogenesis biobank, PWH and controls were prospectively recruited. As GDF-15 is induced by metformin [18, 21], participants using any antidiabetic drugs were excluded.

Baseline samples and data from PWH on ART in the LILAC (CTN PT027) study were included, before intervention [21, 23].

### Laboratory Measurements

Plasma HIV-1 p24 antigen/antibody and confirmation by Western blot were used to establish diagnosis of HIV infection by clinical laboratories of the participating sites. Plasma HIV viral load was quantified with the Abbott Real-Time HIV-1 assay (Abbott Laboratories, Chicago, IL, USA). Plasma samples of study participants were collected in ethylenediaminetetraacetic acid (EDTA) tubes and stored at −80°C. Whole blood CD4 and CD8 T-cell counts were measured using 4-color flow cytometry by clinical laboratories of the participating sites.

From blood samples, plasma and serum were isolated by centrifugation and kept at −80°C until use. Peripheral blood mononuclear cells (PBMCs) were isolated using Ficoll-density gradient (Stemcell Technologies, Vancouver, BC, Canada) and kept in liquid nitrogen until use.

### Measurement of Plasma Biomarkers

Levels of GDF-15, FGF-21, GLP-2, and soluble urokinase plasminogen activator receptor (suPAR) were measured by enzyme-linked immunosorbent assay (ELISA) using commercialized kits (R&D Systems, Minneapolis, MN, USA and Merck, Boston, MA, USA). A MesoScale Discovery multiplex (Rockville, MD, USA) was performed on the CHACS cohort samples, while a 41-plex multiplex from Millipore (Burlington, MA, USA) was used on the HIV pathogenesis biobank samples to quantify plasma levels of cytokines, chemokines, and growth factors. All measures were performed in duplicate as per manufacturers’ instructions.

### Integrated HIV DNA Quantification

Integrated HIV DNA levels were measured in CD4^+^ T cells isolated from PBMCs (Stemcell Technologies). Quantification was performed by nested quantitative polymerase chain reaction (qPCR) using specific HIV long terminal repeat and Alu primers, as we previously described [24]. HIV DNA copy numbers were normalized relative to cell numbers upon simultaneous CD3 DNA amplification with specific primers. All measurements were performed in triplicate. The ACH2 cells carrying one copy of integrated HIV DNA per cell (National Institutes of Health HIV Reagent Program) were used as a standard curve. The limit of detection of these assays was 3 copies per test.

### Intracellular GDF-15 Labeling

Frozen PBMCs were thawed in RPMI medium containing 10% fetal bovine serum (FBS), 100 U/mL of penicillin, and 100 µg/mL of streptomycin (hereafter referred as complete medium), washed with cold phosphate-buffered saline (PBS), and kept at 4°C or on ice until the end of the experiment. Cells were washed with PBS containing 2% FBS 1 mM EDTA (hereafter referred as cytometry buffer). A total of 10^6^ PBMCs per condition were incubated with antibody cocktails for 20 minutes in the dark at 4°C. Panels were designed to identify T-cell and myeloid cells/dendritic cells (DCs). Cells were then washed in cytometry buffer and fixed using 4% paraformaldehyde for 20 minutes at 4°C and then washed in permeabilization buffer (PBS containing 0.1% saponin and 0.1% bovine serum albumin). Intracellular labeling was performed in permeabilization buffer for 45 minutes at 4°C and then washed in permeabilization buffer. Cytometry acquisition was performed on a BD-Fortessa X20, and analysis was performed using FlowJo version 10.9 (gating strategy described in Supplementary Figure 1).

### Statistical Analyses

For all variables, medians with interquartile range (IQR) were calculated. Unpaired comparisons were conducted using *t*-tests or Mann–Whitney *U* tests, as appropriate. Spearman rank correlation test was used to identify associations between 2 quantitative measures. *P* values <.05 were considered significant. Data were analyzed using GraphPad Prism 8.0 (GraphPad Software, San Diego, CA, USA).

### Patient Consent Statement

The CHACS and LILAC (CTN PT027) studies were approved by the research ethics boards of McGill University Health Centre (MUHC), the Centre Universitaire de Montréal, and all participating centers. The HIV pathogenesis biobank has been approved by the research ethics board of MUHC. All participants provided written informed consent. The study was conducted in accordance with the principles of the Declaration of Helsinki.

## RESULTS

### Plasma Levels of GDF-15 Are Elevated in PWH on ART

Participant characteristics are described in Table 1.

**Table 1.** Demographic and Laboratory Results of Participants

Study Groups	PWH in Primary HIV Infection (n = 36)	ART-Naive PWH (n = 42)	PWH on ART (n = 140)	Controls Without HIV (n = 83)	Group Comparisons
Group number	1	2	3	4	
Age, y, median (IQR)	38 (31–44)	38 (33–50)	55 (49.1–62.2)	52 (44–59)	1 vs 3: *P* < .001 1 vs 4: *P* < .001 2 vs 3: *P* < .001
Sex at birth: Female, No. (%)	1 (2.7)	9 (21.4)	13 (9.3)	22 (26.5)	1 vs 4: *P* < .001 3 vs 4: *P* = .02
Sex at birth: Male, No. (%)	35(97.7)	33 (78.5)	127 (90.7)	61 (73.8)	
Race/Ethnicity, No. (%)	32 (88.9)	28 (66.6)	98 (70)	61 (73.5)	1 vs 3: *P* = .002
White	1 (2.7)	7 (16.7)	27 (19.3)	12 (14.5)	
African American	2 (5.5)	7 (16.7)	15 (10.7)	10 (12)	
Latino Asian	1 (2.7)	0 (0)	0 (0)	0 (0)	
CD4 T-cell count, cells/µL, median (IQR)	460 (350–620)	220 (35–345)	588 (408–700)	854 (558–1011)	1 vs 4: *P* < .001 2 vs 4: *P* < .001 2 vs 3: *P* = .03
CD8 T-cell count, cells/µL, median (IQR)	810 (620–1040)	770 (406–1147)	685 (565–804)	425 (281–689)	1 vs 4: *P* = .002 2 vs 4: *P* < .001 3 vs 4: *P* = .006
CD4/CD8 ratio, median (IQR)	0.57 (0.44–0.75)	0.19 (0.06–0.43)	0.74 (0.47–0.77)	1.58 (1.22–2.73)	1 vs 4: *P* < .001 2 vs 4: *P* < .001 3 vs 4: *P* = .06 2 vs 3: *P* < .01
Viral load, log_10_ HIV RNA copies/mL, median (IQR)	4.33 (3.67–4.93)	5.12 (4.42–5.47)	<1.7	NA	1 vs 3: *P* < .001 2 vs 3: *P* < .001
Plasma GDF-15 level, pg/mL, median (IQR)	278.6 (205.7–379.7)	418.4 (359.7–799.7)	881.8 (574.3–1346)	443.4 (315.0–668.4)	See Figure 1

Abbreviations: ART, antiretroviral therapy; GDF-15, growth differentiation factor 15; HIV, human immunodeficiency virus; IQR, interquartile range; NA, not applicable; PWH, people with human immunodeficiency virus.

**Figure 1.**
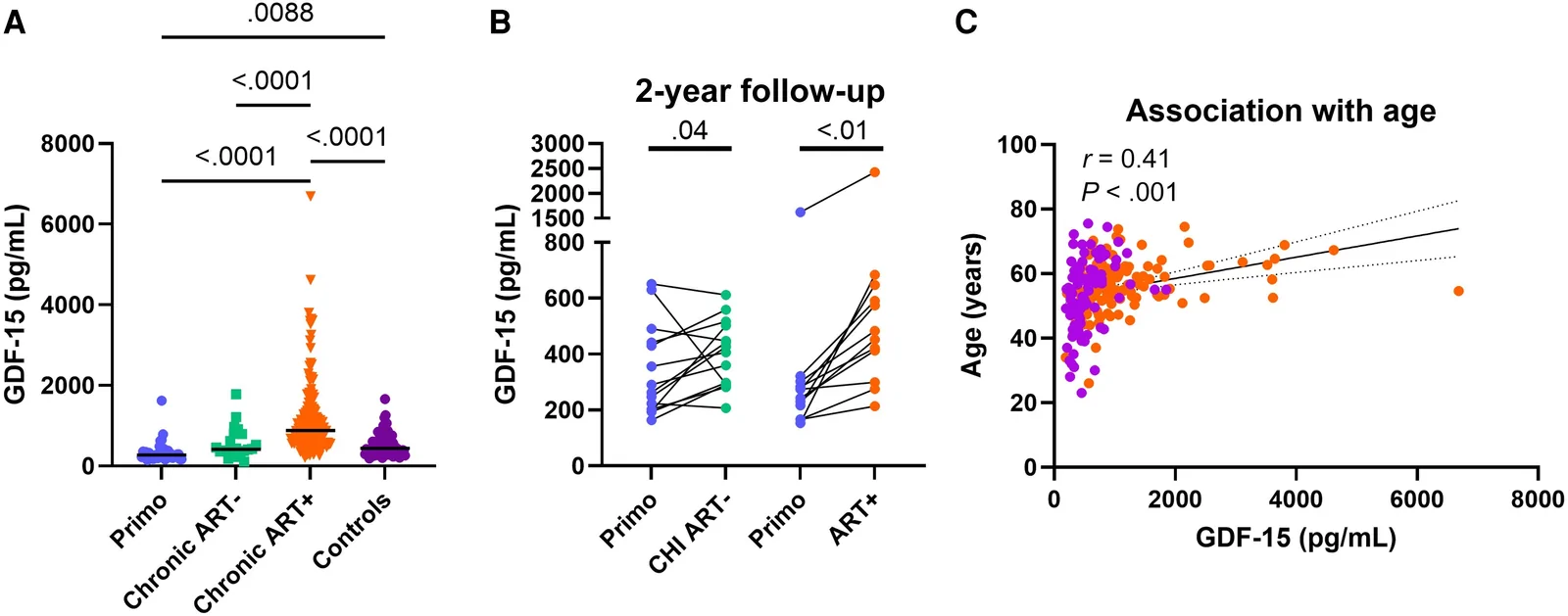
Plasma GDF-15 is elevated in PWH on ART as a marker of accelerated aging. *A*, Plasma GDF-15 levels in PWH in the primary phase of the infection (<6 months after the estimated date of HIV acquisition [Primo]), in people in chronic infection who remained without ART or on ART and controls without HIV with similar age as those on ART. Mann–Whitney test. *B*, Plasma GDF-15 levels at time of diagnosis and 2 years later in those who initiated ART or remained off ART. Wilcoxon test. *C*, Correlation of plasma GDF-15 with age in PWH on ART (orange) and controls (purple). Spearman test. Abbreviations: ART, antiretroviral therapy; GDF-15, growth differentiation factor 15; HIV, human immunodeficiency virus; PWH, people with human immunodeficiency virus.

Plasma levels of GDF-15 were 3.1 times and 2.1 times higher in PWH on ART as compared to ART-naive PWH in primary or chronic infections, respectively (*P* < .001 in both cases), with the naive group being younger. Plasma GDF-15 levels were also 1.98 times higher in PWH on ART compared to uninfected controls (*P* < .0001), with both groups having similar age (Figure 1*A*). Female and male PWH on ART had similar GDF-15 levels (median, 583 [IQR, 409–2226] vs 664 [IQR, 458–1094] pg/mL; *P* = .98). In controls, females and males also had similar GDF-15 levels (476 [IQR, 273–560] vs 386 [IQR, 300–624] pg/mL; *P* = .96). Participants with different type of ART had similar levels of GDF-15 (Supplementary Figure 2).

### Longitudinal Assessment of Plasma GDF-15

We followed a subgroup of PWH diagnosed early (<6 months) after the estimated date of acquisition and followed for 2 years: 12 participants who initiated ART after diagnosis and 14 who remained off ART. Both groups had increased plasma GDF-15 levels at follow-up (Figure 1*B*).

As expected, GDF-15 levels correlated with age in the group of PWH in the primary infection phase (*r* = 0.44, *P* = .007) as well as in ART-treated PWH (*r* = 0.41, *P* < .001; Figure 1*C*). Also, GDF-15 levels correlated with duration of infection (*r* = 0.38, *P* = .008) and time to ART (*r* = 0.31, *P* = .035).

### Plasma Levels of GDF-15 Are Not Associated With Circulating Inflammation Markers

To assess whether persisting inflammation on ART could be involved in GDF-15 induction, we tested correlations between GDF-15 and plasma levels of inflammatory biomarkers assessed using multiplex or ELISA assays in samples available from the CHACS cohort (Table 2). We found positive association between GDF-15 levels and those of CTACK, FLT3L, G-CSF, GLP1, IL-15, IL-1RA, and SDF1a. Together, these biomarkers indicate an association between tissue stress (skin, liver, heart) and activation of myeloid cells. None of the typical biomarkers known to be elevated in PWH on ART (eg, gut permeability markers REG3a, I-FABP, and IL-6 or IL-8) were linked with GDF-15 plasma levels [25].

**Table 2.** Associations Between Plasma Levels of GDF-15 and Several Biomarkers in the Plasma of People With HIV on Antiretroviral Therapy From the CHACS Cohort

Comparison Plasma Levels of GDF-15 vs Plasma	*r*	95% CI	*P* Value (2-tailed)	*P* Value	No.
BDG	0.1038	−.1738 to .3662	.4506	ns	55
BDNF	−0.1176	−.5149 to .3213	.5931	ns	23
bNGF	0.2731	−.1489 to .6109	.1866	ns	25
C-peptide	0.2938	−.1268 to .6249	.154	ns	25
CTACK	**0**.**4438**	**.0468–.7198**	.**0262**	*	25
ENA78	0.000769	−.4049 to .4061	.9971	ns	25
Eotaxin2	−0.1885	−.5518 to .2350	.367	ns	25
Eotaxin3	0.374	−.0823 to .7007	.0949	ns	21
EPO	0.3892	−.0193 to .6864	.0545	ns	25
FGF21	0.2531	−.1699 to .5973	.2222	ns	25
FGF23	0.3466	−.0685 to .6594	.0896	ns	25
FLT3L	**0**.**4746**	**.0856–.7381**	.**0165**	*	25
Fractalkine	0.3046	−.1151 to .6320	.1387	ns	25
G-CSF	**0**.**598**	**.1979–.8273**	.**0054**	**	20
GIP (active)	0.3896	−.0291 to .6919	.0599	ns	24
GIP	0.1077	−.3114 to .4917	.6084	ns	25
GLP1 (inactive)	0.2451	−.1984 to .6052	.2597	ns	23
GLP1	**0**.**5346**	**.1648–.7726**	.**0059**	**	25
GROa	−0.1862	−.5501 to .2373	.373	ns	25
I-309	0.03652	−.3832 to .4437	.8655	ns	24
I-FABP	0.2189	−.0572 to .4640	.1083	ns	55
IFN-γ	0.2877	−.1334 to .6208	.1632	ns	25
IL-12/IL-23p40	0.07231	−.3433 to .4642	.7312	ns	25
IL-15	**0**.**5792**	**.2271–.7974**	.**0024**	**	25
IL-16	−0.16	−.5310 to .2625	.4449	ns	25
IL-17F	−0.1598	−.5628 to .3044	.4889	ns	21
IL-18	0.1954	−.2282 to .5568	.3493	ns	25
IL-1α	0.1723	−.2507 to .5401	.4101	ns	25
IL-1β	−0.1828	−.4339 to .0947	.1816	ns	55
IL-1RA	**0**.**4692**	**.0787–.7349**	.**018**	*	25
IL-22	−0.03436	−.3394 to .2772	.8269	ns	43
IL-23	0.1399	−.3351 to .5582	.5563	ns	20
IL-27	0.05308	−.3602 to .4489	.8011	ns	25
IL-2Rα	0.3669	−.0453 to .6724	.0712	ns	25
IL-37	−0.1031	−.4882 to .3156	.6239	ns	25
IL-6	−0.1085	−.3703 to .1692	.4303	ns	55
IL-7	−0.1054	−.4900 to .3135	.6161	ns	25
IL-8	−0.1139	−.3750 to .1640	.4078	ns	55
IL-9	−0.05154	−.4477 to .3615	.8067	ns	25
Insulin	0.1454	−.2764 to .5202	.4881	ns	25
IP-10	0.1954	−.2282 to .5568	.3493	ns	25
LPS	0.09221	−.1852 to .3560	.5031	ns	55
MCP-1	0.08	−.3364 to .4702	.7038	ns	25
MCP-2	−0.08	−.4702 to .3364	.7038	ns	25
MCP-4	0.04615	−.3662 to .4434	.8266	ns	25
M-CSF	0.3454	−.0699 to .6586	.0908	ns	25
MDC	−0.1746	−.5418 to .2485	.4038	ns	25
MIP-1a	0.3765	−.0443 to .6839	.0698	ns	24
MMP3	0.04782	−.3046 to .3887	.7883	ns	34
MMP9	0.1994	−.1590 to .5113	.2582	ns	34
Proinsulin	0.1138	−.3058 to .4964	.5879	ns	25
sCD14	0.001976	−.4213 to .4245	.9929	ns	23
SDF1a	**0**.**5262**	**.1534–.7678**	.**0069**	**	25
suPAR	**0**.**5876**	**.3749–.7415**	**<**.**0001**	****	55
TARC	−0.1485	−.5225 to .2735	.4788	ns	25
TNF-α	0.00635	−.2669 to .2786	.9633	ns	55
TPO	0.3631	−.0498 to .6699	.0744	ns	25
TRAIL	0.2385	−.1849 to .5872	.251	ns	25
TSLP	0.4079	−.0425 to .7205	.0664	ns	21
VEGF-A	−0.06769	−.4606 to .3473	.7478	ns	25

Abbreviations: CHACS, Canadian HIV and Aging Cohort Study; CI, confidence interval; GDF-15, growth differentiation factor 15; ns, not statistically significant.

Significant correlations are indicated in bold. * *P* < 0.05, ** *P* < 0.01, *** *P* < 0.001, **** *P* < 0.0001.

To confirm that GDF-15 levels were not associated with inflammatory markers, we compared plasma GDF-15 levels with inflammatory biomarkers in 34–44 PWH on ART from the HIV pathogenesis biobank (Table 3). We found an association between GDF-15 and eotaxin levels, a chemoattractant of eosinophils produced upon cellular stress in many tissues and a known marker of aging and cardiovascular risk [26]. GDF-15 levels were also associated with MCP-1 (monocyte chemoattractant protein 1, also known as CCL2), a chemoattractant of monocytes, DCs, and T cells in response to tissue damage [27]. Once again, none of the inflammatory and gut permeability markers were associated with GDF-15 levels. We then assessed whether GDF-15 levels were associated with the frequency of activated CD4 T cells (in a subgroup of participants with available PBMCs) and found no correlation (Table 3).

**Table 3.** Association Between Plasma GDF-15 Levels, Plasma Inflammatory Biomarkers, and T-Cell Activation in the HIV Pathogenesis Biobank

Plasma Levels of GDF-15 vs Biomarkers	*r*	95% CI	*P* Value (2-tailed)	*P* Value	No.
LPS	−0.02629	−.3288 to .2811	.8655	ns	44
BDG	0.2638	−.0529 to .5323	.0914	ns	42
REG-3a	0.2635	−.0532 to .5321	.0917	ns	42
I-FABP	0.2316	−.0830 to .5042	.1351	ns	43
**suPAR**	**0.3723**	**.0758–.6083**	**.0128**	*	**44**
PDGF-AA	−0.2266	−.5321 to .1310	.1974	ns	34
PDGF-AB-BB	0.2735	−.0816 to .5670	.1176	ns	34
RANTES	−0.0515	−.3918 to .3012	.7724	ns	34
G-CSF	0.05504	−.2980 to .3948	.7572	ns	34
EGF	0.2039	−.1544 to .5148	.2475	ns	34
FGF2	−0.01773	−.3628 to .3317	.9207	ns	34
Eotaxin	**0**.**4615**	**.1360–.6971**	.**006**	**	34
TGF-α	−0.1452	−.4689 to .2129	.4125	ns	34
FLT3L	0.2882	−.6570 to .5777	.0983	ns	34
GM-CSF	0.0277	−.3228 to .3715	.8764	ns	34
Fractalkine	−0.1643	−.4840 to .1941	.3531	ns	34
IFN-α2	−0.172	−.4900 to .1865	.3308	ns	34
IFN-γ	0.007801	−.3405 to .3542	.9651	ns	34
GRO	−0.1068	−.4379 to .2498	.5476	ns	34
IL-10	0.1756	−.1829 to .4929	.3206	ns	34
MCP-3	−0.06802	−.4058 to .2861	.7023	ns	34
IL-12p40	−0.1711	−.4894 to .1874	.3333	ns	34
MDC	0.1161	−.2409 to .4455	.513	ns	34
IL-12p70	−0.2163	−.5243 to .1417	.2191	ns	34
IL-13	−0.3126	−.5953 to .0389	.0718	ns	34
IL-15	−0.05664	−.3962 to .2965	.7503	ns	34
sCD40L	−0.02262	−.3671 to .3273	.899	ns	34
IL-17A	−0.2082	−.5181 to .1500	.2373	ns	34
IL-1RA	0.1329	−.2248 to .4591	.4536	ns	34
IL-1α	−0.01437	−.3599 to .3347	.9357	ns	34
IL-9	−0.1434	−.4675 to .2146	.4184	ns	34
IL-1b	−0.05091	−.3913 to .3018	.7749	ns	34
IL-2	−0.1482	−.4713 to .2100	.403	ns	34
IL-4	−0.2421	−.5437 to .1149	.1678	ns	34
IL-5	0.02461	−.3255 to .3688	.8901	ns	34
IL-6	0.02915	−.3215 to .3727	.87	ns	34
IL-7	−0.2335	−.5373 to .1239	.1838	ns	34
IL-8	0.2213	−.1365 to .5281	.2084	ns	34
IP-10	0.1547	−.2036 to .4764	.3825	ns	34
MCP-1	**0**.**3744**	**.0311–.6387**	.**0291**	*	34
MIP-1α	−0.1778	−.4946 to .1807	.3144	ns	34
MIP-1β	0.08039	−.2746 to .4161	.6513	ns	34
TNF-α	0.2834	−.0710 to .5742	.1044	ns	34
TNF-β	−0.1718	−.4899 to .1867	.3314	ns	34
VEGF	−0.2253	−.5311 to .1324	.2002	ns	34
% HLA-DR^+^CD38^+^ CD4 T cells among PBMCs	−0.2637	−.6249 to .1905	.2357	ns	22
% HLA-DR^+^CD38^+^ CD8 T cells among PBMCs	−0.2729	−.6309 to .1810	.2192	ns	22

Abbreviations: CI, confidence interval; GDF-15, growth differentiation factor 15; ns, not statistically significant; PBMC, peripheral blood mononuclear cell.

Significant correlations are indicated in bold. * *P* < 0.05, ** *P* < 0.01, *** *P* < 0.001, **** *P* < 0.0001.

Moreover, plasma GDF-15 levels were not associated with the CD4/CD8 ratio in either cohort (CHACS: *r* = −0.23, *P* = .11, n = 51; HIV pathogenesis biobank: *r* = −0.021, *P* = .88, n = 48).

Overall, we found that GDF-15 levels were not associated with classical inflammatory biomarkers but positively correlated with tissue stress and myeloid cell activation (monocytes, macrophages, DCs, eosinophils) and recruitment during tissue damage.

**Figure 2.**
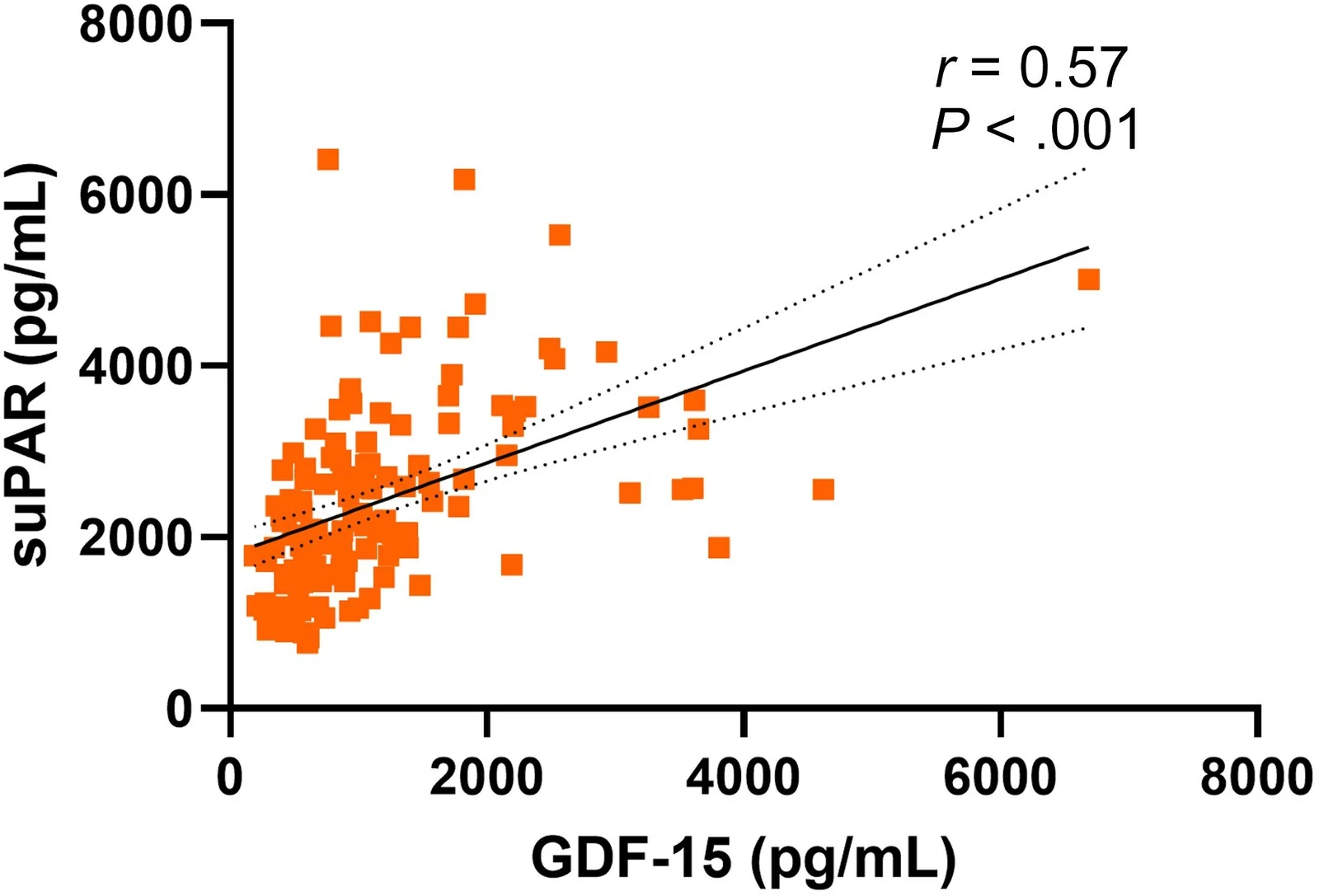
Plasma GDF-15 levels correlate with levels of the comorbidity marker suPAR in people with HIV on ART. Spearman test. Abbreviations: ART, antiretroviral therapy; GDF-15, growth differentiation factor 15; HIV, human immunodeficiency virus; suPAR, soluble urokinase plasminogen activator receptor.

We found a strong correlation between GDF-15 and suPAR plasma levels (Figure 2), both in the CHACS cohort and the HIV pathogenesis biobank (*r* = 0.58, *P* < .001, n = 55 and *r* = 0.37, *P* = .01, n = 44, respectively; Tables 2 and 3). suPAR is commonly expressed in most tissues and is secreted upon tissue damage. Of note, circulating level of suPAR is a validated marker for risk of comorbidities in PWH on ART [28, 29].

However, plasma GDF-15 levels were not associated with circulating levels of mitochondrial stress as assessed by circulating mitochondrial DNA levels (Supplementary Figure 3) [30, 31].

### Plasma Levels of GDF-15 Correlate With Integrated HIV DNA Measures in PWH on ART

**Figure 3.**
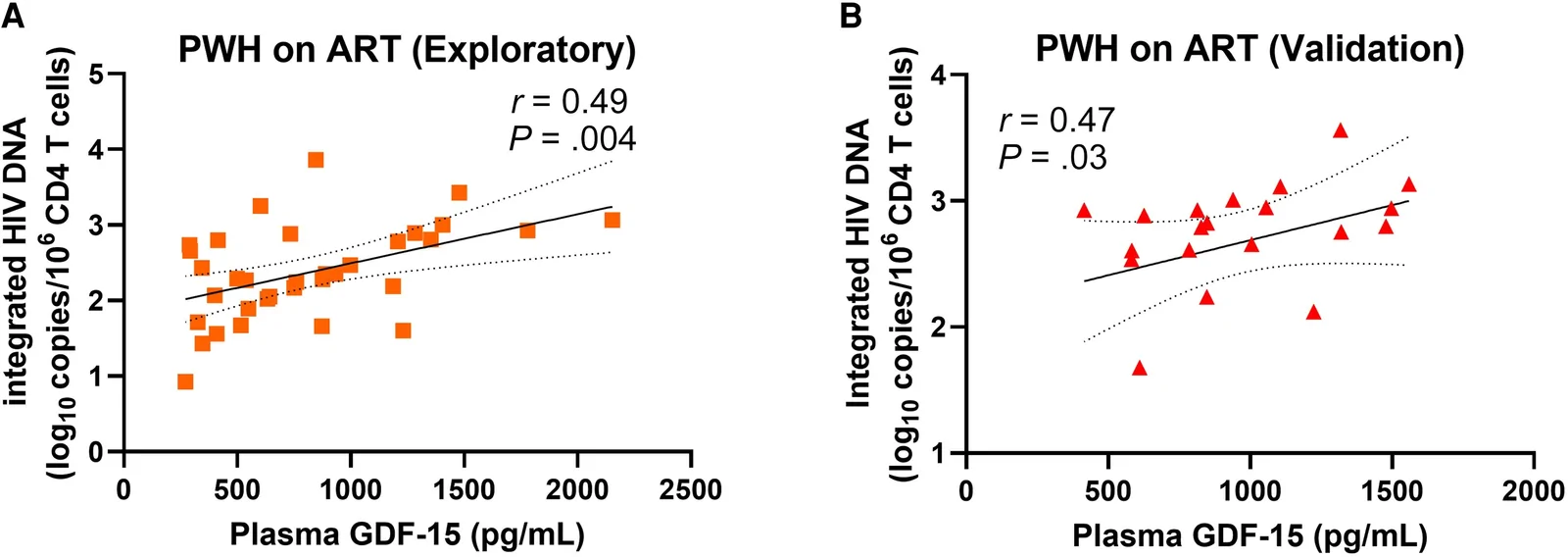
Plasma GDF-15 levels associated with levels of integrated HIV DNA in PWH on ART from 2 cohorts. Plasma GDF-15 levels correlated significantly with proviral HIV DNA levels in CD4 T cells, as assessed by nested quantitative polymerase chain reaction in the CHACS and HIV pathogenesis biobanks as an exploratory study (*A*) as well as in the baseline samples of the LILAC study participants as a validation (*B*). Both studies enrolled PWH on ART for at least 3 years, the LILAC participants having a CD4/CD8 ratio <1. Spearman test. Abbreviations: ART, antiretroviral therapy; CHACS, Canadian HIV and Aging Cohort Study; GDF-15, growth differentiation factor 15; HIV, human immunodeficiency virus; PWH, people with human immunodeficiency virus.

As GDF-15 levels were associated with markers of T-cell proliferation such as IL-15 and FLT3L [32, 33], but not with markers of inflammation, we assessed whether GDF-15 could be linked to HIV persistence. GDF-15 levels, but not those of other biomarkers including IL-6 (*r* = 0.005, *P* = .98), IL-8 (*r* = 0.12, *P* = .58), or suPAR (*r* = −0.05, *P* = .81), positively correlated with levels of integrated HIV DNA in CD4 T cells (Figure 3*A*).

Multivariable analyses showed that the association of GDF-15 with integrated HIV DNA was independent of age and sex of the participants.

To confirm the association between plasma GDF-15 and HIV persistence markers, we compared plasma GDF-15 levels and integrated HIV DNA levels in sorted CD4 T cells from baseline samples from the LILAC pilot study (CTN PT027 [21, 23, 34]). In 21 participants on ART with a CD4/CD8 ratio <1 and detectable levels of integrated HIV DNA levels, we also found a correlation between circulating GDF-15 levels and the frequencies of cells harboring integrated HIV genomes (Figure 3*B*). During subsequent study visits, GDF-15 levels increased due to metformin treatment while HIV DNA levels remained stable [21], then correlation between GDF-15 and HIV DNA was lost (*r* = 0.18, *P* = .46).

### GDF-15 Is Produced by Monocytes in PWH

**Figure 4.**
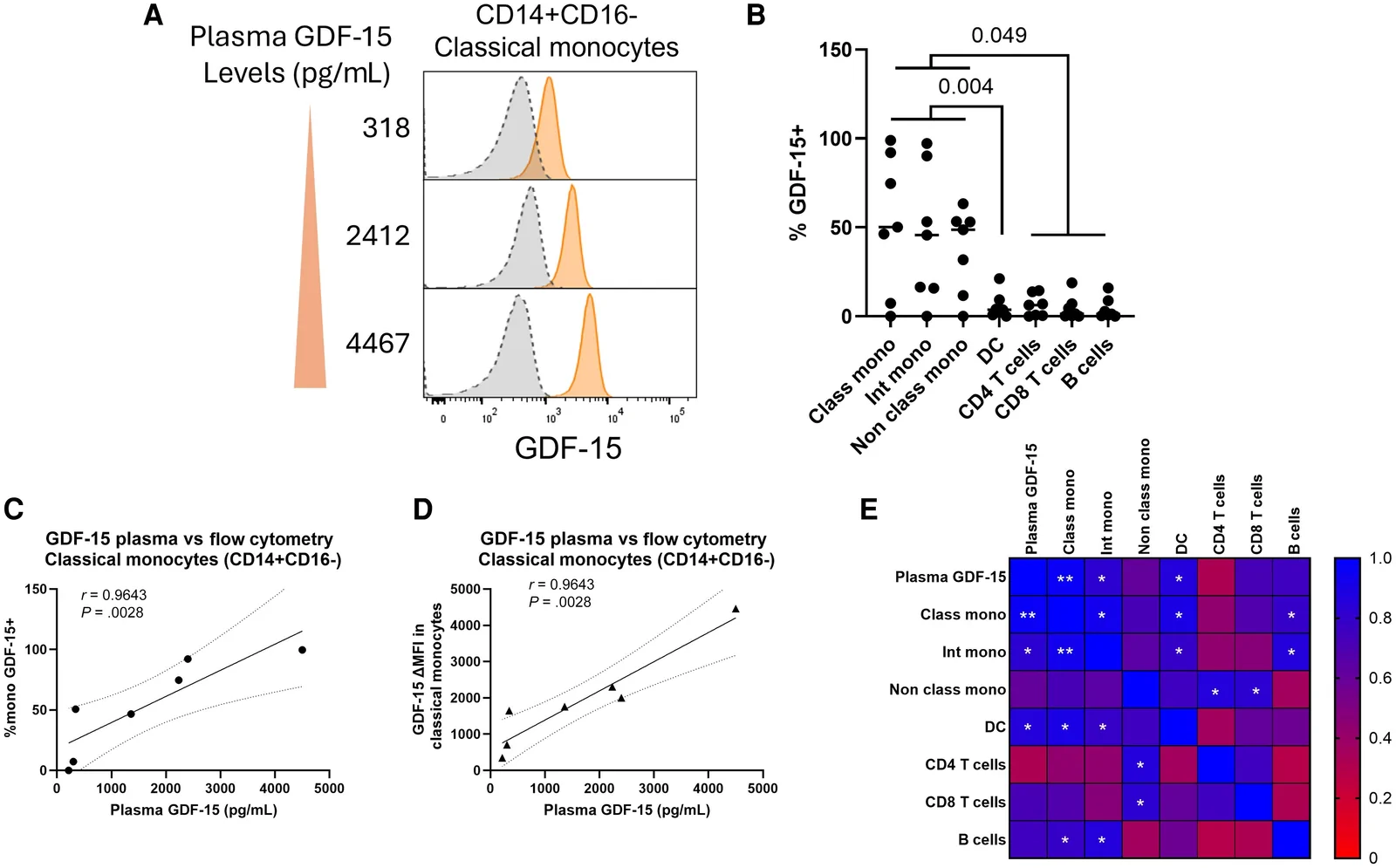
GDF-15 was only produced in monocytes in PWH on ART. *A*, Intracellular GDF-15 levels in classical (CD14^+^CD16^−^) monocytes from 3 representative PWH on ART relative to their plasma levels of GDF-15. Gray histograms show isotype controls. Respective plasma GDF-15 levels are indicated. *B*, Comparison of intracellular GDF-15 levels among different populations in peripheral blood mononuclear cells of PWH on ART (Friedman test). *C* and *D*, Plasma GDF-15 levels correlated strongly with intracellular levels in classical monocytes, as expressed in percentage of GDF-15^+^ cells (*C*) or MFI (*D*). *E*, Correlation matrix of plasma GDF-15 levels and intracellular levels. Plasma levels correlated with frequency of GDF-15^+^ classical and intermediate monocytes, as well as DCs. The *r* values are shown in color scale. *P < .05, **P < .01. Spearman test, n = 7. Abbreviations: ART, antiretroviral therapy; class mono, classical monocyte; DC, dendritic cell; GDF-15, growth differentiation factor 15; HIV, human immunodeficiency virus; Int mono, intermediate monocyte; MFI, median fluorescence intensity; PWH, people with human immunodeficiency virus.

Flow cytometry was used to identify the cellular source of GDF-15. As GDF-15 levels associated with HIV DNA and MCP-1 levels, we looked for GDF-15 in T cells, monocytes, DCs, and B cells. GDF-15 production was observed in classical (CD14^+^CD16^−^), intermediate (CD14^+^CD16^+^), and nonclassical (CD14^dim^CD16^+^) monocytes (Figure 4*A* and 4*B*).

GDF-15 was not produced by naive or memory CD4 or CD8 T cells, nor B cells (Figure 4*A* and 4*B*). Reverse-transcription–qPCR confirmed that GDF-15 mRNA was only found in sorted monocytes (classical, intermediate, and nonclassical) but not in T cells or B cells. On the contrary, as a control, we found that TGF-β was produced by both monocyte types, T and B cells (Supplementary Figure 4).

GDF-15 production in monocytes (expressed as the percentage of GDF-15^+^ cells or as median fluorescence intensity) strongly correlated with plasma GDF-15 levels (Figure 4*C–E*).

Monocytes were sorted and cultivated in complete medium for 7 days. Background production of GDF-15 production was detected in the supernatant. Upon addition of macrophage colony-stimulating factor and granulocyte macrophage colony-stimulating factor to stimulate the differentiation into macrophages, GDF-15 was produced in the supernatant. Addition of lipopolysaccharide (LPS) did not induce GDF-15 secretion by either monocyte or monocyte-derived macrophages, confirming that GDF-15 induction is independent from inflammation (Supplementary Figure 5).

Interestingly, recombinant GDF-15 induced tyrosine phosphorylation at similar levels as TGF-β, suggesting a direct effect on CD4 T cells (Supplementary Figure 6).

## DISCUSSION

In this study, we report elevated circulating levels of GDF-15 in the plasma of PWH on ART. GDF-15 levels were associated with age, comorbidity, and HIV proviral DNA in CD4 T cells, but not with inflammatory markers. Our findings indicate that GDF-15 is produced exclusively by monocytes and is linked with HIV DNA levels in CD4 T cells. In a longitudinal study, we found GDF-15 plasma increasing over 2 years in PWH receiving or not receiving ART. Moreover, GDF-15 levels were not linked with ART regimen in ART-treated PWH. Both findings suggest that GDF-15 is not induced by ART drugs, although this will have to be confirmed in future studies.

GDF-15 has been described as one of the markers of aging [9, 10]. The mechanisms responsible for age-related GDF-15 elevation encompass mitochondrial stress, tissue damage, and senescence. In PWH on ART, accelerated aging has been described [35, 36]. Mitochondrial stress, chronic tissue damage not repaired during ART, and immunosenescence likely converge toward persistent macrophage stimulation and GDF-15 production in PWH on ART. In line with accelerated aging in PWH, we observed a strong correlation between GDF-15, age, and suPAR level, a validated marker for non-AIDS comorbidities in PWH on ART, including cardiovascular disease and cancers. SuPAR is produced upon cleavage of membrane-bound uPAR on immune cells, mostly linked with inflammation and cellular stress [28].

In our cohorts of PWH on ART, despite known persisting levels of inflammation, we found no association between GDF-15 levels and inflammation markers. We explored several markers of innate and adaptive inflammatory pathways, as well as biomarkers of tissue injury, including markers of gut damage, which as we have described do not normalize despite ART [37–39].

Despite not being linked with inflammation, GDF-15 was the only biomarker associated with integrated HIV DNA levels in CD4 T cells. Moreover, GDF-15 levels were not associated with the frequencies of activated (HLA-DR^+^CD38^+^) T cells. These findings indicate that tissue stress and damage could participate indirectly in the maintenance of the pool of infected cells. In PWH on long-term ART, only a few markers such as soluble PD-1 and galectin-9 were associated with the frequencies of HIV-infected cells, most of them being associated with inflammation or immune activation [40–42]. Upon metformin treatment in nondiabetic PWH on ART, GDF-15 increased but HIV DNA levels remained unchanged [18, 21, 34]. Metformin induces mitochondrial stress, thereby stimulating the integrated stress response transcription factor 4 (ATF4) and C/EBP homologous protein (CHOP), which promote GDF-15 transcription [18]. Based on these observations, we can speculate that the pathways leading to metformin-induced GDF-15 production are different from the one linking GDF-15 to HIV DNA levels. As we found no link between mitochondrial mass or mitochondrial DNA damage and GDF-15 levels [31, 43], the mechanism leading to GDF-15 production in PWH on ART will have to be further explored.

Despite GFRAL not being expressed by CD4 T cells (proteinatlas.org), GDF-15 appeared to induce signalization in CD4 T cells. It is possible that GDF-15 signals through CD48, the only putative receptor of GDF-15 that could be expressed on T cells. CD48 triggering has been shown to act as a co-stimulatory molecule, influencing T-cell, T regulatory cell, and natural killer cell activity [12]. The role of the GDF-15–CD48 pathway should be further studied in the context of chronic infection.

In the blood, we found that GDF-15 is exclusively produced by monocytes. In vitro, GDF-15 was only produced upon differentiation of monocytes into macrophages. These results corroborate previous findings of GDF-15 production in macrophages in mouse models [44–46]. However, LPS stimulation of monocytes did not induce GDF-15 secretion, nor did it increase production in macrophages. To our knowledge, this is the first demonstration of GDF-15 production in monocytes and secretion upon differentiation into macrophages independently of inflammation. In animal models of acute bacterial or viral infection, GDF-15 was shown to be necessary for survival and tolerance of infection through a regulatory mechanism [47]. Moreover, GDF-15 production in macrophages was shown to be profibrotic [48, 49]. In PWH on ART, such tolerogenic mechanisms could be involved by decreasing T-cell cytotoxic function, contributing to the maintenance of HIV-infected CD4 T cells. In animal models, adipose tissue macrophages were shown to produce GDF-15 [45].

Our study has limitations as measurements were exclusively performed in circulating blood. The link between tissue stress and GDF-15 production should be confirmed in tissue biopsies. Moreover, our study is mostly correlative and the direct link between GDF-15 production, risk of non-AIDS comorbidities, and HIV persistence should be further studied.

In a phase 2 clinical trial, GDF-15 inhibition with the monoclonal antibody ponsegromab led to increase weight in patients with metastatic non-small cell lung cancer, pancreatic cancer, or colorectal cancer–associated cachexia [50]. Molecular mechanisms leading to GDF-15 production in PWH should be further studied, together with the potential use of GDF-15 blockade in an effort to reduce the size of HIV reservoirs and decrease the comorbidity risk of ART-treated PWH.

## CONCLUSIONS

Monocytes produced GDF-15 independently of inflammation and GDF-15 levels were linked with marker for non-AIDS comorbidities and HIV persistence in PWH on ART. These observations suggest that tissue damage and cellular stress could indirectly promote HIV persistence in infected cells. New therapeutic strategies aiming at promoting tissue repair, decreasing GDF-15 and senescence should be evaluated in PWH on ART.

## Supplementary Material

jiag202_Supplementary_Data

## Supplementary Data

Supplementary materials are available at *The Journal of Infectious Diseases* online (http://jid.oxfordjournals.org/). Supplementary materials consist of data provided by the author that are published to benefit the reader. The posted materials are not copyedited. The contents of all supplementary data are the sole responsibility of the authors. Questions or messages regarding errors should be addressed to the author.
