# Supplementary material for: Plasma GDF-15 Levels Are Associated With HIV Reservoir Markers Independently of Inflammation in People With HIV on Antiretroviral Therapy
Source: J Infect Dis. 2026 Apr 9;234(2):e374–84. doi: 10.1093/infdis/jiag202 (PMC13537152; doi:10.1093/infdis/jiag202)
Supplement: Supplementary file 1 [file jiag202_supplementary_data.docx]

**Isnard *et al***

**Supplementary data:**

**Supplementary Figure 1:** gating strategy for identification of monocytes, DC, T and B-cells in PBMCS for sorting and intracellular GDF-15 assessment.

**Supplementary Figure 2:** Comparison of GDF-15 plasma levels in PWH with different antiretroviral regimens.

**Supplementary Figure 3:**  comparison of plasma GDF-15 levels with circulating levels of mitochondrial DNA, quantifying D-LOOP and ND4 genomic sections.

**Supplementary figure 4:** RT-qPCR assessment of GDF-15 (left) and TGF-β (right) mRNA levels in flow cytometry sorted cells.

S**upplementary figure 5:** Quantification of GDF-15 or IL-6 in the supernatant of monocytes or monocyte-derived macrophages in the presence of M-CSF and GM-CSF. Cells were stimulated for a total of 7 days, and LPS or PBS as control were added during the last 18hours.

**Supplementary Figure 6. GDF-15 induced signaling in isolated CD4 T-cells.**

Recombinant GDF-15 or TGF-β were added at 200ng/ml on isolated CD4 T-cells for 30 min at 37^o^C, then intracellular levels of phopho-tyrosine (Tyr) were assessed by flow cytometry. 2 independent experiments on cells from 2 donors each. Show are th4e normalized MFI values relative to unstimulated cells. ANOVA test.
